# A Deep-Learning-based 3D Defect Quantitative Inspection System in CC Products Surface

**DOI:** 10.3390/s20040980

**Published:** 2020-02-12

**Authors:** Liming Zhao, Fangfang Li, Yi Zhang, Xiaodong Xu, Hong Xiao, Yang Feng

**Affiliations:** Research Center of Intelligent System and Robotics, Chongqing University of Posts and Telecommunications, Chongqing 400065, China; zhaolm@cqupt.edu.cn (L.Z.); liffang123@gmail.com (F.L.); xxd@cqupt.edu.cn (X.X.); fy1222111@163.com (Y.F.)

**Keywords:** continuous casting, surface defects, 3D imaging, neural network, deep learning, defect detection

## Abstract

To create an intelligent surface region of interests (ROI) 3D quantitative inspection strategy a reality in the continuous casting (CC) production line, an improved 3D laser image scanning system (3D-LDS) was established based on binocular imaging and deep-learning techniques. In 3D-LDS, firstly, to meet the requirements of the industrial application, the CCD laser image scanning method was optimized in high-temperature experiments and secondly, we proposed a novel region proposal method based on 3D ROI initial depth location for effectively suppressing redundant candidate bounding boxes generated by pseudo-defects in a real-time inspection process. Thirdly, a novel two-step defects inspection strategy was presented by devising a fusion deep CNN model which combined fully connected networks (for defects classification/recognition) and fully convolutional networks (for defects delineation). The 3D-LDS’ dichotomous inspection method of defects classification and delineation processes are helpful in understanding and addressing challenges for defects inspection in CC product surfaces. The applicability of the presented methods is mainly tied to the surface quality inspection for slab, strip and billet products.

## 1. Introduction

In recent years, with the advent of the industrial 4.0 enterprises undergoing transformation and upgrading manufacturing processes, continuous casting (CC) as a main solidification process for molten steel has been widely popularized to produce metal semi-finished products [1]. In the iron and steel industry with the maturity of CC technology, hot charging and direct rolling (HC-DR) as an energy-efficient production pattern is currently experiencing rapid development [2,3]. Technically, to implement HC-DR, the defect-free CC products will undoubtedly be an essential prerequisite [4,5]. Although the technical objectives to be improved have been identified, no manufacturer in the world has reported one-hundred percent defect-free CC semi-products manufacturing technology in such a complex and systematic setting [6]. Therefore, complementary technologies such as automatic nondestructive examination (NDE) for CC products surface quality evaluation have become essential in the promotion of HC-DR [7,8]. This is an advisable method to eliminate flaw segments according to accurate NDE evaluation results [9]. Machine vision (MV) in NDE combined with AI algorithms is becoming a burgeoning method which can perform with a fast response, a high signal-to-noise ratio and a strong anti-jamming capability [10,11] compared with ultrasonic, eddy current and other contact methods. The MV merits make it more competitive in harsh environment application like CC manufacturing field [12,13]. On the other hand, MV-based 3D optical metrology has gradually demonstrated superiority, such as [14,15,16] stereoscopy triangulation (mm), interferometry (nm), con-focal vertical scanning, and fringe projection (um). ArcelorMittal Corp. developed a conoscopic holography rangefinders system tested in ACERALIA Crop. (Spain). The Cognex Corp. in the US developed a SmartView detection system that applied to a wide variety of surface defects inspection tasks. Elkem Corp. in Norway and Honeywell Corp. in the United States conducted infrared and visible-light MV detection methods [17]. Xu et al. [18], based on MV technology, carried out extensive research on CC slab and rolled strip surface defects inspection. To obtain effective 3D defects shapes, Zhao et al. [19] combined line array CCD and area array CCD imaging methods and devised the informative image scanning method. As a fast developing subfield of machine learning, multilayer perceptron convolutional neural networks combined with deep learning(CNN-DL) strategies in MV inspection field have shown state-of-the-art performance [20]. CNN-DL methods do not require laborious hand-craft features for classifier design [21] and as a branch of ANN, they make the complex function approximation feasible by learning a deep nonlinear network. He Di et al. [22] trained a classifier for strip defects recognition based on convolutional auto-encoder (CAE) and a devised semi-supervised Generative Adversarial Networks. To overcome the trivial image pre-processing and feature extraction process, Wangzhe Du et al. [23] presented an X-ray defect detection system based on the Feature Pyramid Network and a data augmentation method for model generalization training. Veitch-Michaelis et al. [24] studied the 3D cracks recognition method through the combination of morphological detection and SVM classifier. Hongwen Dong in Northeastern University proposed a pyramid feature fusion and a global context attention network for pixel-wise detection of surface defect in the industrial production process [25]. Fatima A. Saiz et al. [26] reported a deep-learning based automatic defects recognition system in which CNN was utilized in the model design, which achieved an outstanding classification rate. CNN-DL strategies need to make full use of training datasets and learning algorithms to make the detection results relatively stable. Therefore, they generally require a large number of training samples as input. In high-noise environments, MV based-intelligent inspection methods as mainstream schemes have been successfully applied in the CC products line, although the accuracy and mechanism of the AI algorithm require in-depth research with the improvement of application requirements.

**Figure 1:**
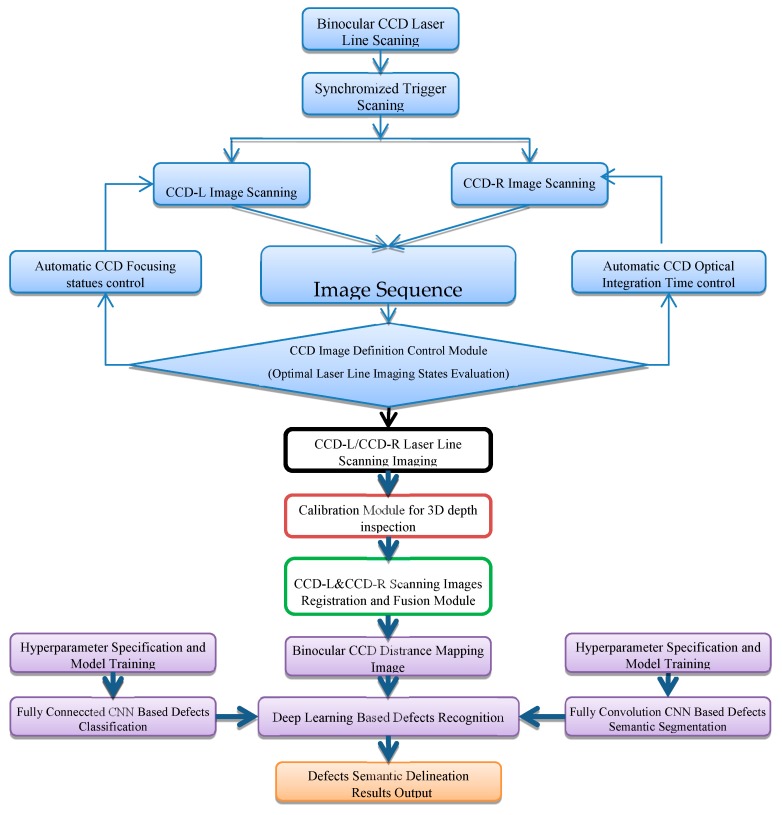
The scheme of binocular CCD-based 3D image deep-learning CC products surface defects inspection.

In the CC production line, with the improvement of quality requirements, the defect depth has become a significant factor, which, especially for the CC slab, sometimes may cause potential security problems. In other words, some defects can be ignored or repaired by the follow-up finishing process if the depth of the defects does not exceed a certain value. Furthermore, conventional optical imaging 2D inspection methods are susceptible to high-temperature radiation interference. In this work, we refer to the entire defects inspection process as two separate steps: recognition and delineation, and based on our previous work in [6], a novel two-step defects inspection strategy was presented by devising a fusion deep CNN model (fully connected CNN with fully convolution CNN). The entire scheme, as shown in Figure 1, was implemented by the devised flexible binocular 3D quantitative inspection deep-learning system (3D-LDS). In this system, unlike traditional inspection methods the 3D depth point cloud mapping images will be feed into 3D-LDS. Furthermore, a region proposal method was designed using 3D-LDS ROI location that can effectively suppress redundant candidate bounding boxes in a real-time defects recognition process. Systematically a 3D-LDS-based CNN-DL strategy was attempted for CC products surface defects inspection that allows a feasible method of AI algorithms and powerful ROI recognition and delineation strategies to be further studied in industrial applications.

## 2. An Improved 3D Image Scanning System

### 2.1. Optimal Image Laser Scanning Method

**Figure 2:**
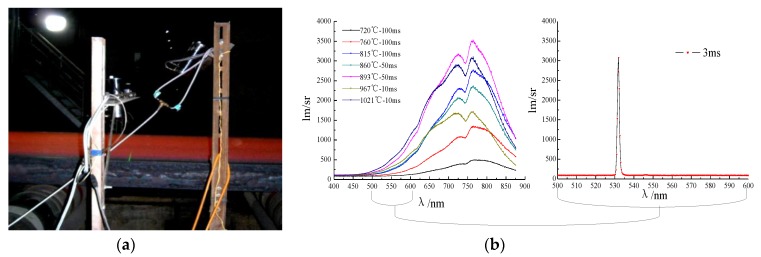
The spectral radiation intensity at different temperatures in slab surface and light intensity distribution of the laser stripe. (**a**) High temperature spectrum measurement; (**b**) Spectral intensity comparison.

In image-based ROI inspection methods, it is a prerequisite for the imaging sensor to be able to capture objects informatively and adjust imaging parameters adaptively as the peripheral environment changes. Therefore, the 3D-LDS as a structured light assistant active imaging system needs a laser stripe with a maximal color contrast and the most homogeneous gray-level. Namely, the imaging sensor should be set to an appropriate optical integral time (OIT) and focus status. When it comes to a rigid system architecture, the focus status can be fixed as the imaging distance and imaging depth of field (DOF) are constants. However, the automatic OIT controlling method needs to be focused on if imaging sensor works in an unstable high-temperature radiation environment. According to the Planck theorem [27], we took the CC production as an blackbody and assumed that its surface emissivity is equal to 1. While T > 500 ℃ (like the CC slab roughly varied between 600 ℃ to 900 ℃ when it comes out of the second cooling area), the visible red-light radiation can be sensed by unaided eyes. We tested the optical spectrum radiation interference in different temperatures in hot CC slab surface from 720 ℃ to 1021 ℃, as in shown in Figure 2. We can observe the regular patterns of light strength distribution with different OIT and object surface temperatures. The experiments present a quantitative guidance for determining laser luminous wavelength and controlling the imaging sensor’s parameters. In 3D-LDS, to minimize radiation interference, we selected a 532 nm green laser emitter. On the one hand, it can ensure that the CCD sensor is in the imaging spectral sensitive range and on the other hand, it can avoid high-temperature radiation interference as much as possible. We can observe that the radiation intensity of the laser stripe at 3 ms is easily distinguishable from the hot slab surface (1000℃) at the integral time of 10 ms.

On the basis of the light radiation principle, we presented an improved method to determine threshold $T_{L}$ in 3D-LDS, which allows the CCD cameras to scan the laser stripe precisely without being interfered by a high temperatures radiation. Based on the CCD imaging principle, theoretically, the objects luminance can be formulated as follows [28]:

(1) $$E_{0}={\left(\frac{n^{\prime }}{n}\right)}^{2}K\pi L\sin^{2}U^{\prime },$$

where n and $n^{\prime }$ denote, respectively, the refractive index in object space and image space, K is the optical system transmittance, L is the light luminance, and $U^{\prime }$ represents the image aperture angle. Supposing that the laser reflected luminance can be expressed by $L^{\prime }=\rho E$, where E represents laser transmitter luminance and ρ is the reflectivity (0<ρ<1), then, the diffuse reflection of the laser stripe on the object surface can be formulated by

(2) $$E_{0}^{\prime }={\left(\frac{n^{\prime }}{n}\right)}^{2}K\pi \rho E\sin^{2}U^{\prime },$$

**Figure 3:**
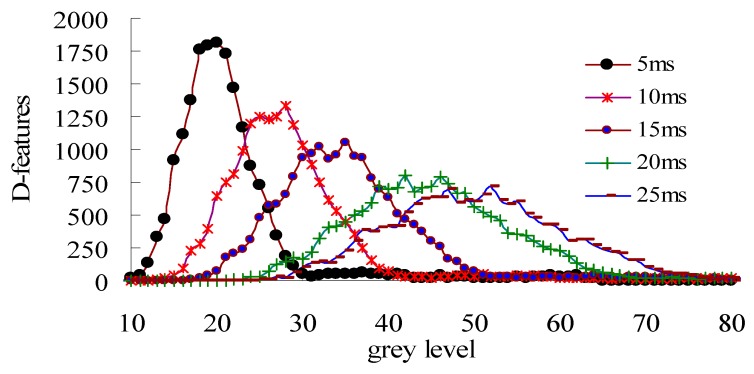
Laser stripe shape and intensity distribution counts at different CCD integral times.

Apparently, as shown in Figure 3, quantitatively determining the best color distance between the slab surface and the laser stripe depends on the threshold at the optimal light integration time [29]. It also shows that in the figure, the laser stripe shape is easily extracted when the light intensity is concentrated.

Therefore, $T_{L}$ can be found by the following method. Firstly, we convert the 24-bit color image into gray level directly by assigning R=G and B=G. While the CCD sensor’s images have pixel levels of [1,..T..,L], let $n_{i}$ and N denote the number of pixels at level i and the total number in one frame, then the $T_{L}$ should be between the $\mu_{b}$ and $\mu_{f}$ [30]:

(3) $$\left\{ \begin{array}{l} \mu_{b}=\sum_{i=1}^{T}ip_{i}/\omega_{b}=\mu (T)/\omega (T)\\ \mu_{f}=\sum_{i=T+1}^{L}ip_{i}/\omega_{f}=\frac{\mu_{t}-\mu (T)}{1-\omega (T)} \end{array}\right.,$$

where $p_{i}=n_{i}/N$, $\mu (T)=\sum_{i=1}^{T}ip_{i}$, $\mu_{t}=\sum_{i=1}^{L}ip_{i}$, $\omega_{t}=\sum_{i=1}^{T}p_{i}=\omega (T)$, $\omega_{f}=\sum_{i=T+1}^{L}p_{i}=1-\omega (T)$. The variances of the foreground of the laser stripe and the background are formulated as follows:

(4) $$\left\{ \begin{array}{l} \sigma_{b}^{2}=\sum_{i=1}^{T}{\left(i-\mu_{b}\right)}^{2}p_{b}/\omega_{b}\\ \sigma_{f}^{2}=\sum_{i=T+1}^{L}{\left(i-\mu_{f}\right)}^{2}p_{f}/\omega_{f} \end{array}\right.,$$

Based on the Otsu and CCD imaging definition variance evaluation function, the optimal scanning threshold T can be determined by the following discriminate criterion measure:

(5) $$f(T)=\sigma_{B}^{2}(T)/\sigma_{t}^{2},$$

where $\sigma_{B}^{2}(T)=\omega_{b}{\left(\mu_{b}-\mu_{t}\right)}^{2}+\omega_{f}{\left(\mu_{f}-\mu_{t}\right)}^{2}$ denotes the classes variance and $\sigma_{t}^{2}=\sum_{i=1}^{L}{\left(i-u_{t}\right)}^{2}p_{i}$ represents the current frame total variance. In fact, optimal $T_{o}$ can be computed by searching the threshold interval [1,..T..,L] to meet the requirement:

(6) $$T_{L}=\underset{1\leq T\leq L}{max}\sigma_{B}^{2}(T),$$

**Figure 4:**
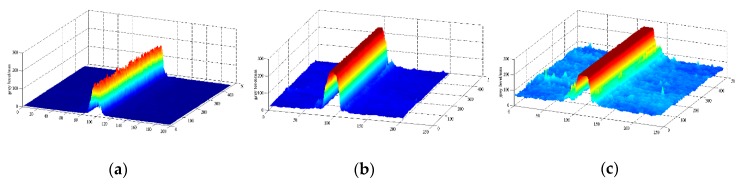
Laser stripe imaging features at different CCD imaging states. (**a**) Short CCD integral time; (**b**) Optimal laser imaging; (**c**) Long CCD integral time.

Figure 4 displays the laser imaging results that Figure 4b is the most convenient shape for data processing through experiments under optimal imaging states.

### 2.2. System Construction

**Figure 5:**
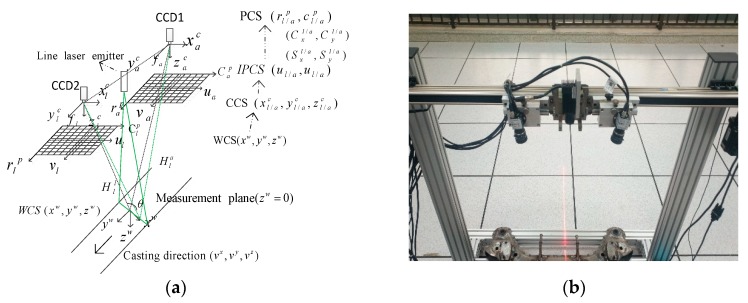
The schematic illustration of the binocular CCD laser image scanning system. (**a**) The schematic working principle diagram; (**b**) The corresponding devised experimental system.

To implement the deep learning 3D inspection method and create a reliable detection system to meet the special requirements, we devised an improved experimental system based on our previous research. Figure 5a is the schematic principle of the devised binocular CCD laser image scanning system. Figure 5b is the corresponding experimental system devised that we updated from our previous multi-source CCD imaging system in the literature [6]. The previous system mainly utilized the traditional inspection methods, and the 3D laser scanning system just played auxiliary role in defect location. In the new 3D-LDS system, the integrity of defects can be captured properly without the line scanning CCD. In this system, we employed two MERCURY CCD cameras (model: MER-500-14GC-P) and the lens model selected was M0814-MP2. Here, the deep learning defects recognition process was conducted on the fusion image from the two imaging sensors. In this system, the two laser scanning images were overlaid informatively by a registration method and this process is a rigid transformation of rotation and translation. Once the system calibration was completed, the imaging parameters between the two CCD cameras were settled. Notice that the applicability of the proposed experimental system is not tied to CC products surface defects inspection exclusively. 

**Figure 6:**
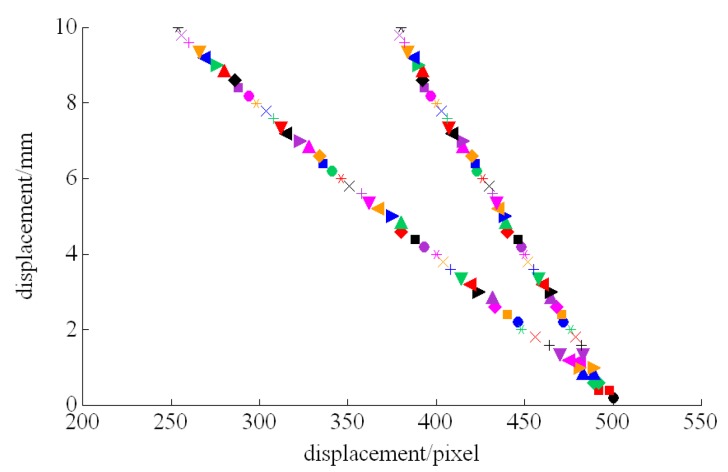
The influence of different oblique angles on detection accuracy and sensitivity (left:$\theta /2=60^{{}^{\circ}}$ and right: $\theta /2=45^{{}^{\circ}}$ ).

In the system, the 3D images pixels (12-bit) are indirectly mapped from the calibrated laser triangulation strategies (the metric is millimeter). Therefore, the image ROI was reconstructed by converting the 3D distance point cloud of the object surface. From the experiments in Figure 6, we can visually observe that the system can change its detection accuracy and sensitivity for depth information by finely adjusting θ according to the detection requirements. Generally, CNN-DL model training requires a large number of labeled examples. We utilized the angular fine adjustment to acquire different scanning images for the testing samples as an auxiliary data augmentation method. The depth of variation was explicitly added to the training samples. Based on this method, we also used the typical variation, including changes in contrast, rotations and translations. Deep-learning is extremely data-hungry and performance grows only logarithmically with the amount of data used. This is one of main limitations that the field is currently facing.

## 3. CNN-DL Inspection Method Design in 3D-LDS

### 3.1. CNN Networks Design in 3D-LDS

**Figure 7:**
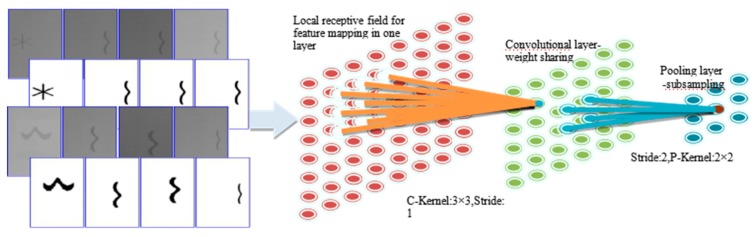
The convolution and pooling process in CNNs for locality perception and parameters sharing mechanism.

In neural networks, a neuron is the fundamental unit that takes a bias $w_{0}$ and a weight vector $\omega =(w_{0},\ldots w_{n})$ as parameters to a decision model: $f(x)=h(\omega^{T}x+w_{0})$ where h(x) is a non-linear activation function. More complex nonlinear mapping is usually based on the combination of lots of neurons that are arranged in layers. Commonly, a single layer network can be expressed as a linear combination of *N* individual neurons [31]:

(7) $$\tilde{f(x)}=\sum_{i=0}^{N-1}v_{i}h(w^{T}x+w_{0,i}),$$

where the trainable parameters for this network can be summarized as ($v_{0}$, $w_{0,0}$, $w_{0}$,..., $v_{N}$, $w_{0,N}$, $w_{N}$). Appropriate parameters can decrease the ideal function and its approximation: $\left|f(x)-\tilde{f}(x)\right|$. Theoretically, any function can be approximated using a single layer network only if we give a large number of neurons and have the proper parameters within the same compact set that the network can be trained. The more layers (deeper networks) the network creates, the stronger the networks’ modeling capacity. However, the deeper the number of layers, the more challenging it is to train the network parameters. In recent years, deep learning technology has been widely used in many fields, especially the proposed convolutional and pooling payers make the model have a robust ability to extract local and macro characteristics. In Figure 7, the convolutional and pooling process in DL networks achieved locality perception and parameter-sharing mechanism, which dramatically reduce the amount of model training parameters. In addition, the End-to-End training strategy makes the feature extraction-selection and classifier design integrated in a streamlined process. The hand-crafting features are no longer required while everything is learned by the network model based on a data-driven mode.

**Figure 8:**
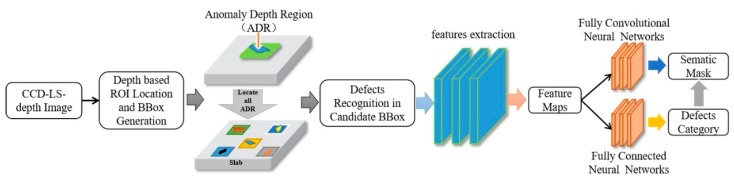
Defects recognition and delineation process based on deep CNN modeling mechanism.

Based on the end-to-end training mechanism, we built a complete deep neural network model in 3D-LDS. As shown in Figure 8, we devised a dichotomous defects inspection strategy that includes two steps and a two-branch deep neural network for defects types classification (recognition) and ROI delineation. In the overall inspection process, the input images mapped from the laser triangulation were finally converted to a predication map and a classification label. The proposed methodology is helpful in understanding and addressing challenges for CC production surface inspection. In the recognition process, 3D point cloud images in 3D-LDS was utilized to locate the defect positions accurately according to the depth detection results. Through the initial location of the possible ROI(defects) the candidate bounding box(BBox) will be generated, which we define this process as depth based ROI initial location and BBox generation. In the last two steps, the BBox will be classified by fully connected neural networks and the defects types will be output in images level, and the prediction map in pixel-wise will be output in fully convolutional neural networks for delineation.

**Figure 9:**
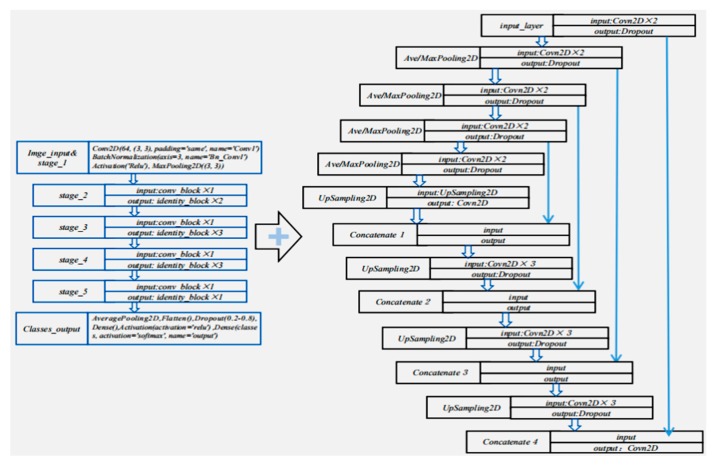
The schematic network architectures for defects recognition and ROI delineation.

A significant characteristic of DL strategies is the automatic feature learning for data representations through an end-to-end training process. To realize the two-step defects recognition and delineation in 3D-LDS, we constructed a novel network architecture by integrating the blocks of Resnet [32] and Unet [33]. The aim is to take advantages of the deep CNN merits in classifier design and fuzzy ROI delineation. Thereinto, ResNet were designed to enable training of very deep networks due to the residual block is introduced. Ronneberger’s full convolution idea is a breakthrough towards automatic image segmentation. In fact, the ROI segmentation can be expressed as an auto encoder and decoder process. It consists of a contracting and an expanding branch and enables multi-resolution analysis. Figure 9 indicates the schematic network architectures for defects classification(recognition) and ROI segmentation (delineation). A novel idea here is the devised multi-model-based recognition and delineation that in the defects inspection process the system will according to the input images size automatically select different training models. Usually, the detected candidate ROI will have different sizes to reduce the computational complexity in 3D-LDS only the BBox will be input into system as shown in Figure 8. In the experimental testing process, we trained five different sizes of BBoxes for classifier and delineation DL models (input sizes: 32*32,48*48,64*64,80*80,128*128), the candidate depth ROI based BBoxes will be resized to one of the 5 sizes according to its size proximity. Note that the images will be reconstructed after the recognition and delineation are finished because the real location in CC products surface will be predicted through the system measurement calibration parameters.

### 3.2. Model Training Strategies in 3D-LDS

**Figure 10:**
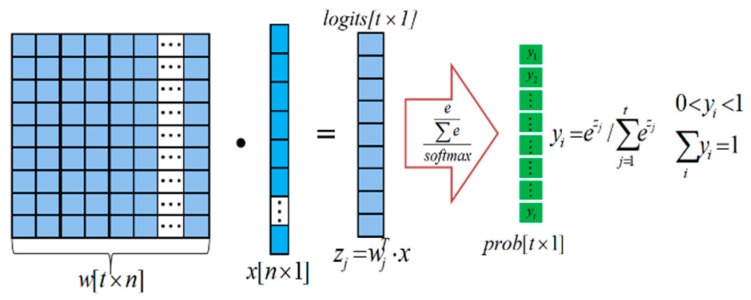
The system multi-classification method for different defects types in training and testing process.

Generally, a CNN network consists of convolutional layers, pooling layers, full connection layers and loss layers, etc., among them, the algorithms in the full connection layer and yjr loss layer are basic parts of the network. CNN based recognition methods have been widely used in image analysis fields. CNN based modeling capability is gradually strengthened owing to the improvement of loss function and optimization algorithm in model training process. In this work, as shown in Figure 10 we utilized softmax function to train multi-classification model [34]:

(8) $$P(y=j|z^{\left(i\right)})=\phi_{softmax}(z^{\left(i\right)})=\frac{e^{z^{\left(i\right)}}}{\sum_{k=1}^{t}e^{z_{k}^{\left(i\right)}}},$$

We can see that the range of this function value is defined in [0,1], where, $z=w_{0}x_{0}+w_{1}x_{1}+\cdots +w_{n}x_{n}=\sum_{i=0}^{n}w_{i}x_{i}=w^{T}x$, t represents the total number of defects categories, w is the weight vector, x is the feature vector of a training sample, and $w_{0}$s the bias unit. $z_{k}$ denotes the value of the output of class k, in the experimental process we basically tested five classifications of defects for transversal cracks, longitudinal cracks, star cracks, hole-shaped defect and others respectively. The softmax function computes the probability that the current training sample $x^{\left(i\right)}$ belongs to class j given the weight and net input $z^{\left(i\right)}$. Therefore, we compute the probability $\left(y=j|x^{\left(i\right)};w_{j}\right)$ for each class label in j=1,….k. Note that the normalization term in the denominator causes the whole class probabilities sum up to one under the assumption that the training samples are independent of each other.

Based on the softmax function we can introduce the softmax *loss* as formulated as below:

(9) $$L=-\sum_{j=1}^{T}y_{j}logs_{j},$$

Here, $s_{j}$ is the j-th value of the output vector s from softmax function, which indicates the probability that the testing sample belongs to the j-th category. $y_{j}$ is a vector of 1*T that only the value of the position corresponding to the real label is equal to 1. Therefore, this formula actually has a simpler form when j is the real label that points to the current sample:

(10) $$L=-logs_{j},$$

Next, we can give the concept of cross entropy which it is formulated as below:

(11) $$E=-\sum_{j=1}^{T}y_{j}logp_{j},$$

Here, *cross entry* is equal to softmax loss while the input $p_{j}$ of cross entry is the output of *softmax.* In our work, we set the activation function as softmax in dense layer. Based on the above discussion, we can define the function of the optimization to minimize (or maximize) the loss function E in training process. Basically, gradient descent is one of the most popular algorithms to perform optimization and up to now the most common way to optimize neural networks. Moreover, there are three basic variants of gradient descent which differ in how much data we use to compute the gradient of the objective function, which include [35] batch gradient descent (GGD), stochastic gradient descent (SGD) and mini-batch gradient descent (MBGD). In fact, there are some challenges need to be solved in allusion to the above three optimization methods. However, these methods are often used to test the effectiveness of the network training process. We will not pay too much attention to these issues because of the focus of this paper. In these experiments, we utilized the adaptive moment estimation (Adam) optimization to compute adaptive learning rates for network parameters. Adam keeps an exponentially decaying average of past gradients similar to momentum besides storing an exponentially decaying average of past squared gradients like Adadelta and RMSprop [36]. Adam prefers flat minima in the error surface and the decaying averages of past and past squared gradients $m_{t}$ and $v_{t}$ are computed separately as follows [37]:

(12) $$\begin{array}{l} m_{t}=\beta_{1}m_{t-1}+(1-\beta_{1})g_{t}\\ v_{t}=\beta_{2}v_{t-1}+(1-\beta_{2})g_{t}^{2} \end{array}$$

where $m_{t}$ *and* $v_{t}$ are estimates of the first moment and the second moment of the gradients respectively, if the $m_{t}$ *and* $v_{t}$ are initialized as vectors of 0, they counteract these biases by computing bias-corrected first and second moment estimates:

(13) $${\overset{\wedge }{m}}_{t}=\frac{m_{t}}{1-\beta_{1}^{t}},\;{\overset{\wedge }{v}}_{t}=\frac{v_{t}}{1-\beta_{2}^{t}},$$

Therefore, based on the bias-corrected estimates, the Adam gradient update rule is generated as below:

(14) $$\theta_{t+1}=\theta_{t}-\eta \frac{{\overset{\wedge }{m}}_{t}}{\sqrt{\overset{\wedge }{v_{t}}}+\varepsilon },$$

The authors propose default values of 0.9 for $\beta_{1}$, 0.999 for $\beta_{2}$, and $10^{-8}$ for ε.

### 3.3. Experimental Results Analysis

**Figure 11:**
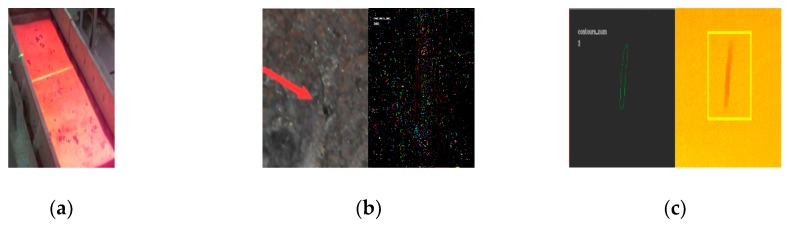
Searching for ROI in optical image and laser scanning (depth) image in 3D-LDS. (**a**) CCD image (Object depth:3mm); (**b**) Searching results for ROI; (**c**) Laser scanning image and search results for ROI.

Due to the all-pervading oxide scales on CC products surface have similar characteristics with real defects, especially in 2D images while it is processed by imaging processing algorithms. We call it pseudo defects interference in inspection process as presented in Figure 11b. The steel plate displays confusing ROI with a crack and also some other outliers. This will make ROI extraction very challenging even in room temperature. In Figure 11b we clustered the ROI and finally found 1400 candidate ROIs. Figure 11c shows the laser scanning image for the Figure 11b, by the same way the counterpart of Figure 11b given by Figure 11c contains 3 candidate ROIs. Therefore, the selective patches given by the location of the candidate ROI will be computed and returned by the recognition model in 3D-LDS. Basically, region proposal algorithms are often employed to identify prospective objects in an image such as the proposed methods of objectness, randomized prim or selective search and so on. In this paper we referred to the region proposal method but devised a more effective way by referring to the laser scanning images depth location as given in Figure 11c. The candidate bounding boxes for defects recognition will be proposed and resized to the closest image patch for recognition.

**Figure 12:**
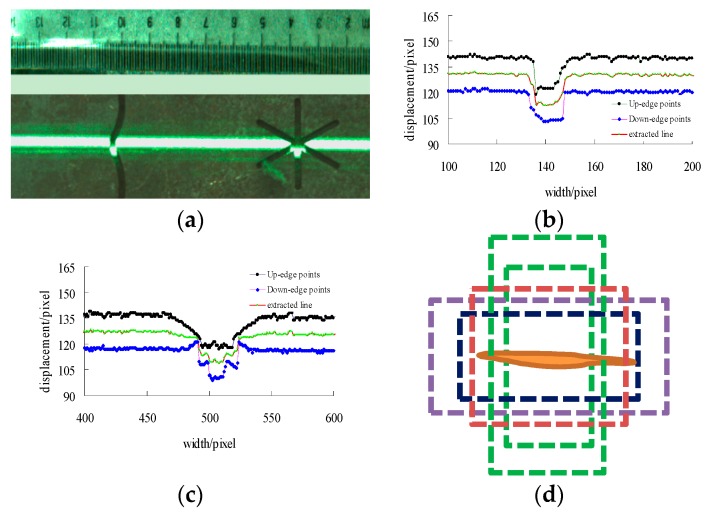
The candidate ROI location in different areas. (**a**) The testing sample defects; (**b**) ROI location in linear defect; (**c**) ROI extraction in star defect; (**d**) The candidate bounding box.

Figure 12 denotes the ROI depth location method. For abnormal depth areas we only extract the centroid line as the position depth values and 3D image reconstruction in scanning process. Figure 12a is the artificial defect that for convenience of calculation we made some samples of different depths and sizes for four defect types and others (made randomly). Figure 12b,c are the laser location process that pixels offset reflected on the image. Figure 12d is the ROI depth based candidate bounding box generation method.

**Figure 13:**
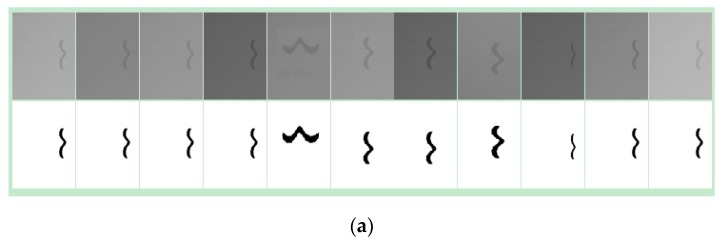

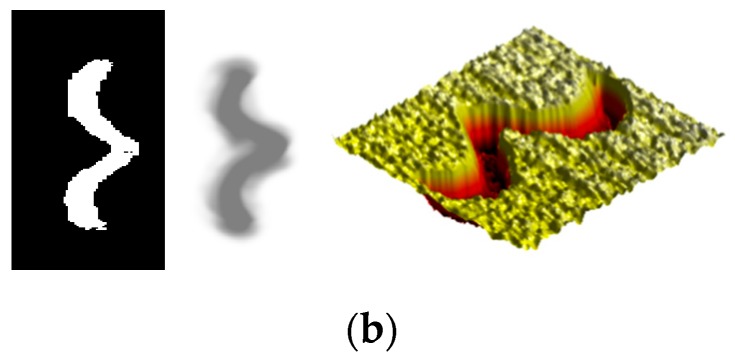
Experimental results for training data sets and predicted reconstruction images. (**a**) Training samples for crack based on the devised data augmentation in 3D-LDS; (**b**) Predication map (left) with its binary image (middle) and the 3D visualization (right) for the inspected L-crack defect.

Figure 13a shows the training samples for L crack generated in 3D-LDS in different scanning angles, distances and optical integral times. The labels (ground truth) in second row are mainly delineated manually and generated by an interactive method to ensure accuracy. In this work, the data augmentation strategy was utilized, the parameters we used for generating a new image are as follow:rotation_range,translation_shift_range,zoom_range and blur operation. Roughly, the training and testing data sets were split in 7:3 separately from different original data. Figure 13b shows the testing results that actually is a reconstructed image from the mapping pixels’ prediction values. We can set a different classification number for the softmax *function* to obtain different output. However, the final binary image will be segmented by a fixed threshold.

In the 3D-LDS defect inspection process, there is a sensitive parameter: the radius of the candidate bounding box(BBox-R), which determines the size of the ROI relative to the size of BBox. Generally, in order to ensure the candidate BBox includes the ROI accurately. We can set a relatively large radius to locate the ROI. However, this will lead to regional imbalances (RI) and consequently, bring about two main issues, especially in full convolutional networks training and the testing process:

(i) In the training process, the RI problem will make CNN-DL model training more challenging to converge and become time consuming because of the unbalance of positive and negative pixel samples.
(ii) In the testing process, RI defects always get undesirable segmentation results by automatic strategies due to the inaccurate positioning by traditional bounding box.

Table 1 is the testing results for five types of defects, thereinto, L-110(440) means the type is longitudinal cracks and training and testing samples are 440 and 110 respectively. T means transverse crack, S denotes star shape defects and H means hole defects. To facilitate the quantitative analysis, we employed image segmentation evaluation methods to test validation in delineation step that includes dice coefficient (DICE), false positive (FP), false negative (FN) and mean hausdorff distance(M-HD). Dice is twice the area of overlap between ground truth(A) and prediction(B) divided by the total number of pixels in both regions [38]:

(15) $$Dice=\frac{2\left|A\cap B\right|}{\left|A\right|+\left|B\right|}\times 100\%,$$

Dice value ranges from 0 to 1 with 1 signifying the greatest similarity between the predicted and truth.

We also used the FP and FN to give us an overall understanding for the predicted results. Because both of the FP and FN are errors in data reporting in which a test result improperly indicates presence of a condition. In general, we will get under segmentation results if the FP is greater than FN and and vice versa. Meanwhile, we utilized M-HD to check the predicted boundary as it is sensitive to it. However, we use the *mean* computing way instead of the *max* method to prevent isolated point noise interference:

(16) $$d_{H}(X,Y)=mean\{d_{XY},d_{YX}\}=mean\{\underset{x\in X,y\in Y}{maxmind(x,y)},\underset{y\in Y,x\in X}{maxmind(x,y)}\},$$

**Figure 14:**
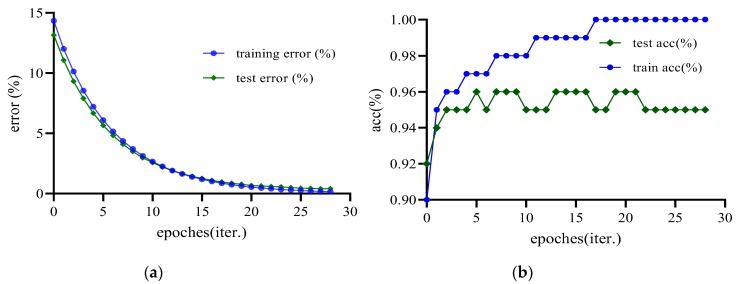
Validation on model training and testing; (**a**) Training and testing errors; (**b**) ACC results on training and testing data sets.

In the model training process, we utilized the basic quantitative quality indicators ACC to validate the system:

(17) ACC=(TP+TN)/(TP+TN+FP+FN),

ACC reflects the classifier’s overall prediction correctness that TP represents the number of observations correctly assigned to the positive class. TN is the number of observations correctly assigned to the negative class. FP denotes the number of observations assigned by the model to the positive class. FN is the number of observations assigned to the negative class, which in reality belong to the positive class. Figure 14 is the validation process for training error and testing error. Table 1 shows the quantitative experimental results that we used the extra FP and FN to get feedback for over-segmentation and under-segmentation so that we can adjust the model parameters.

In allusion to the running time, we tested on the computer with two GPU cards: GEforce GTX 1080 and GEforce RTX 2080Ti, the 2080Ti was used to do the delineation and tested on the maximum BBox(320 × 320). It can perform 15 image segmentation tasks per second that meets the CC production online detection process. With regard to the image scanning speed, we tested image size: 1200 × 600 (the selected CCD cameras is 14fps in full resolution: 2592 × 1944). The system can finish 45fps laser scanning because only laser ROI will be processed in the image. Therefore, the casting speed should be less than 0.8 m/min if the scanning spacing is 0.3 mm. Actually, in real application, the high-performance image workstation or multi-machine distributed processing is preferred. The quantitative experimental results are given in Table 1. 

## 4. Conclusions and Future Work

In this paper, an improved binocular vision-based 3D laser image scanning deep-learning system (3D-LDS) was established for CC products surface evaluation. The main work is as below:

1) An optimal CCD laser image scanning method was designed in different high-temperature experiments.
2) In allusion to defects precise recognition, we proposed a novel region proposal method based on the 3D ROI initial location that can effectively suppress redundant candidate bounding boxes generated by pseudo-defects in a real-time recognition process.
3) To improve the inspection accuracy, a deep CNN architecture combined fully connected networks (for defects classification) and fully convolutional network (for defects delineation) was proposed to robustly make the whole inspection methodology defined as a two-step process.
4) The applicability of the presented methods is mainly tied to the surface quality inspection for slab, strip and billet products etcetera. Systematically, A 3D-LDS deep learning system is devised for CC products surface quality evaluation that allows an automatic way of AI algorithms to be applied to the MV inspection field in modern industries.

Future work: Based on the experimental analysis, it is found that the optimization of network architecture is a long-term job. There is no unified network model for different detection tasks and targets. Therefore, it is essential to conduct field experimental studies to improve and construct a more robust network architecture especially for the defects classification network. The aim is to solve the common over-fitting problem of current networks and to reduce the dependence on data source quality in model training process. Furthermore, the improvement method of optimization algorithm for deep CNN model training should be further studied through the deep neural network mechanism research in the specific application context. In the following work, we will carry out field experiments and application research in the continuous casting production line. 

## Figures and Tables

**Table 1:** Experimental quantitative results.

Type-Total(AUG)	DICE	FP	FN	M-HD
L-110(440)	0.93	0.05	0.08	0.32
T-121(484)	0.87	0.04	0.21	0.17
S_82(328)	0.85	0.03	0.24	0.07
H_102(408)	0.82	0.05	0.21	0.29
Others-12	0.83	0.07	0.25	0.33

## References

[1] Thomas B.G. (2018). Review on modeling and simulation of continuous casting. Steel Res. Int..

[2] Santos C.A., Spim J.A., Garcia A. (2003). Mathematical modeling and optimization strategies (genetic algorithm and knowledge base) applied to the continuous casting of steel. Eng. Appl. of Artif. Intell..

[3] Popa E.M., Kiss I. (2011). Assessment of Surface Defects in the Continuously Cast Steel. Acta Tech. Corviniensis-Bull. Eng..

[4] Song K., Yan Y. (2013). A noise robust method based on completed local binary patterns for hot-rolled steel strip surface defects. Appl. Surf. Sci..

[5] AI Y., Xu K. (2013). Surface Detection of Continuous Casting Slabs Based on Curvelet Transform and Kernel Locality Preserving Projections. Int. J. Iron Steel Res..

[6] Zhao L., Ouyang Q., Chen D., Udupa J.K., Wang H., Zeng Y. (2014). Defect detection in slab surface: A novel dual charge-coupled device imaging-based fuzzy connectedness strategy. Rev. Sci. Instrum..

[7] Ouyang Q., Zhao L.M., Ma F.J., Zhang L.Z. (2011). Experiment study of surface defects in continuous casting using developed laser scanning system. Ironmak. Steelmak..

[8] Zhao L.M., Ouyang Q., Chen D.F., Wen L.Y. (2011). Surface Defects Inspection Method in Hot Slab Continuous Casting Process. Ironmak. Steelmak..

[9] Hsu C.Y., Huang J.W., Kang L.W., Weng M.F. Fast image stitching for continuous casting steel billet images. Proceedings of the IEEE International Conference on Consumer Electronics-Asia (ICCE-Asia).

[10] Hsu C.Y., Kang L.W., Lin C.Y., Yeh C.H., Lin C.T. (2014). Vision-Based Detection of Steel Billet Surface Defects via Fusion of Multiple Image Features.

[11] Zhao Q.-J., Cao P., Tu D.-W. (2014). Toward intelligent manufacturing: Label characters marking and recognition method for steel products with machine vision. Adv. Manuf..

[12] Ouyang Q., Zhang L.Z., Zhao L.M., Zhang X.L., Chen D.F. (2011). Experimental study on quantitative surface defect depth detection based on laser scanning technology in continuous casting. Ironmak. Steelmak..

[13] Ai Y., Xu K. (2012). Feature extraction based on contourlet transform and its application to surface inspection of metals. Opt. Eng..

[14] Xu K., Yang C., Zhou P., Liang J. (2010). 3D Detection Technique of Surface Defects for Steel Rails Based on Linear Lasers. J. Mech. Eng..

[15] He Y., Song K., Meng Q., Yan Y. (2019). An End-to-end Steel Surface Defect Detection Approach via Fusing Multiple Hierarchical Features. IEEE Trans. Instrum. Meas..

[16] Jiang Z., Zhang W., Cui L. (2018). Research of three dimensional laser scanning coordinate measuring machine. MATEC Web of Conferences.

[17] Marc G., Li G. Inspection of Aircraft Engine Components Using Induction Thermography. Proceedings of the IEEE Canadian Conference on Electrical & Computer Engineering (CCECE).

[18] Peng Z., Ke X., Chaolin Y. (2018). Surface defect recognition for moderately thick plates based on a SIFT operator. J. Tsinghua Univ. (Sci. Technol.).

[19] Zhao L., Zhang Y., Xu X., Xiao H., Huang C. (2016). Defect inspection in hot slab surface: Multi-source CCD imaging based fuzzy-rough sets method. Proc. SPIE 9971, Applications of Digital Image Processing.

[20] Ferguson M., Ak R., Lee Y.T.T., Law K.H. Automatic localization of casting defects with convolutional neural networks. Proceedings of the IEEE International Conference on Big Data (Big Data).

[21] Lee J.H., Oh H.M., Kim M.Y. Deep learning based 3D defect detection system using photometric stereo illumination. Proceedings of the International Conference on Artificial Intelligence in Information and Communication (ICAIIC).

[22] Di H., Ke X., Peng Z., Dongdong Z. (2019). Surface defect classification of steels with a new semi-supervised learning method. Opt. Lasers Eng..

[23] Du W., Shen H., Fu J., Zhang G., He Q. (2019). Approaches for improvement of the X-ray image defect detection of automobile casting aluminum parts based on deep learning. NDT E Int..

[24] Veitch-Michaelis J., Tao Y., Walton D., Muller J.P., Crutchley B., Storey J., Paterson C., Chown A. Crack Detection in “As-Cast” Steel Using Laser Triangulation and Machine Learning. Proceedings of the 13th Conference on Computer and Robot Vision (CRV).

[25] Dong H., Song K., He Y., Xu J., Yan Y., Meng Q. (2020). PGA-Net: Pyramid Feature Fusion and Global Context Attention Network for Automated Surface Defect Detection. IEEE Trans. Ind. Inform..

[26] Saiz F.A., Serrano I., Barandiarán I., Sánchez J.R. A Robust and Fast Deep Learning-Based Method for Defect Classification in Steel Surfaces. Proceedings of the International Conference on Intelligent Systems (IS).

[27] Ouyang Q., Zhao L.M., Wen L.Y., Bai C.G. (2011). Simulation study on radiative imaging of pulverised coal combustion in blast furnace raceway. Ironmak. Steelmak..

[28] Fabijanska A., Sankowski D. (2009). Computer vision system for high temperature measurements of surface properties. Mach. Vis. Appl..

[29] Roy M., Seo D., Oh S., Yang J.W., Seo S. (2017). A review of recent progress in lens-free imaging and sensing. Biosens. Bioelectron..

[30] Otsu N. (1979). A threshold selection method from gray-level histograms. IEEE Trans. Syst. Man Cybern..

[31] Shin H.C., Roth H.R., Gao M., Lu L., Xu Z., Nogues I., Yao J., Mollura D., Summers R.M. (2016). Deep convolutional neural networks for computer-aided detection: CNN architectures, dataset characteristics and transfer learning. IEEE Trans. Med. Imaging.

[32] He K., Zhang X., Ren S., Sun J. Deep residual learning for image recognition. Proceedings of the IEEE Conference on Computer Vision and Pattern Recognition.

[33] Olaf R., Fischer P., Brox T. (2015). U-net: Convolutional networks for biomedical image segmentation. International Conference on Medical Image Computing and Computer-Assisted Intervention.

[34] Jiang M., Liang Y., Feng X., Fan X., Pei Z., Xue Y., Guan R. (2018). Text classification based on deep belief network and softmax regression. Neural Comput. Appl..

[35] Jiwoong I.D., Tao M., Branson K. (2016). An empirical analysis of the optimization of deep network loss surfaces. arXiv.

[36] Ruder S. (2016). An overview of gradient descent optimization algorithms. arXiv.

[37] Kingma D.P., Ba J. (2014). Adam: A method for stochastic optimization. arXiv.

[38] Shamir R.R., Duchin Y., Kim J., Sapiro G., Harel N. (2019). Continuous dice coefficient: A method for evaluating probabilistic segmentations. arXiv.

